# Comparison of the nutritional composition and calculated Nutri-score classifications of the Dutch food retail supply in 2018 and 2020

**DOI:** 10.1017/S136898002400154X

**Published:** 2024-10-10

**Authors:** Elly Steenbergen, Elisabeth HM Temme

**Affiliations:** Department of Prevention, Lifestyle and Health, National Institute for Public Health and the Environment, Bilthoven, The Netherlands

**Keywords:** Nutri-Score, Front-of-Pack nutritional label, Incentive, Food reformulation, The Netherlands

## Abstract

**Objective:**

In the Netherlands, reformulation strategies have been established for several years, whereas Nutri-Score was implemented in 2024. Besides being a helpful tool for consumers to make healthier food choices, Nutri-Score also aims to stimulate food reformulation by food manufacturers. The present study investigates whether changes in food composition could have led to different calculated Nutri-Score classifications.

**Design:**

Food compositions and Nutri-Score classifications were calculated using the updated Nutri-Score algorithm. Food groups with the largest change in the distribution of Nutri-Score classifications were analysed in-depth by plotting frequency distributions and calculating median contents for nutrient contents that relatively changed the most in 2020.

**Setting:**

Food composition data were available from the Dutch Branded Food database in 2018 (*n* 38 295) and 2020 (*n* 48 091).

**Participants:**

Not applicable.

**Results:**

In general, median nutrient contents and calculated Nutri-Score classifications were similar for 2018 and 2020. The median sugar and SFA contents were lower for some food groups (e.g. breakfast cereals, meat preserves, sweets and sweet goods) in 2020 compared to 2018. The median SFA content for meat preserves and sweets and sweet goods was relatively low in Nutri-Score classification A ascending towards higher median content in Nutri-Score classification E.

**Conclusions:**

Although food reformulation was not substantial in the Dutch food retail supply in 2018 and 2020, some differences in Nutri-Score classifications were observed. When implemented, Nutri-Score may encourage food manufacturers to increase their reformulation efforts. Repeated monitoring of food compositions and Nutri-Score classifications is recommended to establish reformulation efforts by food manufacturers.

An unhealthy diet is one of the major risk factors for non-communicable diseases^(1)^. To improve consumers’ health, several policy strategies are recommended by the WHO, such as the promotion of healthy food environments through food reformulation and the use of Front-of-Pack nutritional labels (FOPNL)^(2)^.

In the Netherlands, such strategies have been or are being established by the government. Agreements between the government and the industry to reformulate foods were reached under the so-called National Agreement to Improve Product Composition, which ran from 2014 to 2020. This agreement included maximum limits for the salt, saturated fat and sugar content for specific types of foods sold in supermarkets^(3)^. It was shown that the agreements led to improvements in food compositions of several foods such as filet americain (–13 % salt), red cabbage with apples (–20 % salt) and soft drinks (–14 % sugar)^(4)^. Yet, according to an impact assessment for a daily consumption, it led to small improvements with respect to the daily intake of salt (–0·5 g) and sugar (–7·5 g)^(5)^. Expanding the scope to include all types of processed foods and with the intention to expedite the improvements that were agreed upon, the agreement is followed by the new system called the National Approach to Product Improvement which started in 2022. With this new approach, the Dutch government further stimulates manufacturers to reformulate foods by providing criteria for food composition on food group level with goals set up for 2030^(6)^.

Additionally, on 25 April 2023, the start of the implementation of the FOPNL Nutri-Score was officially announced in the Netherlands. From 1 January 2024, Nutri-Score may be voluntarily used by food manufacturers in the Netherlands^(7)^. Nutri-Score was developed by Santé publique France, the French National Public Health Institute, and elected as the official national FOPNL in France in 2017. It has since been adopted in a growing number of European countries^(8)^. As of 2021, a transnational governance structure was established with representatives from the Netherlands, Belgium, France, Germany, Luxembourg, Spain and Switzerland. The International Steering Committee of Nutri-Score facilitates the use and implementation of Nutri-Score and decides on improvements proposed by the International Scientific Committee of Nutri-Score. The International Scientific Committee of Nutri-Score assesses potential improvements of the Nutri-Score algorithm based on scientific literature and includes requests of stakeholders and the synergy with national dietary guidelines. The Technical Committee of Nutri-Score studies technical requests and provides technical support to food manufacturers and retailers^(9)^. On 29 June 2022 and 1 February 2023, the updated Nutri-Score algorithms for solid foods and beverages respectively proposed by the Scientific Committee were accepted by the Steering Committee^(10–12)^.

The Nutri-Score algorithm was based on the Food Standards Agency Nutrient Profiling System from the United Kingdom and uses a numerical outcome variable as well as a categorical outcome variable. The algorithm allocates points to energy and nutrient contents and calculates a final sum of points (numerical variable) which corresponds to the five letter and colour-coded classifications (categorical variable), ranging from A (dark green) to E (dark orange), according to the nutritional quality of the food. Negative points are allocated to unfavourable components (energy, salt, SFA and sugar) and positive points are allocated to favourable components (protein, fibre, fruits, vegetables and legumes). Allocation of points as well as the calculation of the final sum of points slightly differ between general solid foods, beverages, and fats, oils, nuts and seeds, as well as for foods within the food groups such as red meat and beverages containing non-nutritive sweeteners^(10–13)^.

Studies using the updated Nutri-Score algorithm are yet limited. However, in Norway, the updated Nutri-Score algorithm was found to be mostly in line with the food-based dietary guidelines^(14)^. Besides, using the previously applied algorithm, Nutri-Score was found to be an effective tool in discriminating between healthier and less healthier packaged foods in several European countries^(15–19)^. Besides being a helpful tool for consumers to make healthy food choices, Nutri-Score’s second objective is to stimulate food reformulation by food manufacturers. A recent study in the Netherlands using theoretical scenarios with the previously applied algorithm has shown that Nutri-Score could be an incentive for food reformulation^(17)^. However, analyses have not yet been carried out using actual food composition data to calculate Nutri-Score classifications using the recently updated Nutri-Score algorithm.

The present study aims to gain insight into food compositions and to calculate Nutri-Score classifications of foods sold in Dutch supermarkets in 2018 and 2020. Secondly, the present study aims to investigate whether changes in the compositions of these foods could have led to different Nutri-Score classifications.

## Methods

### Data preparation

For the analyses carried out in the present study, food composition data of branded foods in 2018 and 2020 were used. These composition data were obtained from the Dutch Branded Food database. This database contains food label information of branded foods (both private labels and manufacturer brands) and covers approximately 75 % of the Dutch retail food market^(20)^. For the purpose of this study, only processed foods were included and categorised into food groups (2018: *n* 38 324; 2020: *n* 48 170) as defined according to the Dutch RIVM Reformulation Monitor 2020^(4)^. The food group dairy and plant-based foods were further divided into two groups of solid foods and beverages as the Nutri-Score is calculated using different algorithms for solid foods and beverages (see online supplementary material, Supplementary Table 1)^(10,11,13)^.

Foods were excluded from the analyses when data were missing on the algorithm components energy, salt, SFA, sugar and/or protein (2018: *n* 21; 2020: *n* 49). Depending on food group, missing values were relatively more present for fibre content (2018: *n* 11 595; 2020: *n* 17 739) since the declaration of fibre content is not mandatory. Therefore, the mean fibre content was calculated (of the foods for which fibre content was available) and imputed for the missing values of the fibre content in 2018 and 2020 at food group level (see online supplementary material, Supplementary Table 1). Foods were excluded when their food group size was smaller than ten items (2018: *n* 8; 2020: *n* 38). As data on the content of fruits, vegetables and legumes (%) were not available from the database, estimations per food group were made and used for the analyses (see online supplementary material, Supplementary Table 1). For the allocation of points for the protein content of red meat and for the use of non-nutritive sweeteners in beverages, the name and ingredient list of foods were searched for relevant terms.

### Data analyses

The median and interquartile range of energy and nutrient contents were calculated at food group level for 2018 and 2020. Points were allocated according to nutrient contents and were used to calculate Nutri-Score classifications of foods. The distribution of the final sum of points and the corresponding Nutri-Score classifications (dark green A to dark orange E, in %) were calculated using food composition data of foods for 2018 and 2020. The most recent algorithm of the Nutri-Score was used, which has been approved by the International Steering Committee of Nutri-Score for solid foods (on 26 July 2022)^(10)^ and beverages (on 1 February 2023)^(11)^ as recommended by the International Scientific Committee of Nutri-Score. Nutri-Score guidelines as provided by Santé publique France were followed for the analyses^(13)^.

Changes in the distribution of Nutri-Score classification between 2018 and 2020 were expressed in percentage points for each classification. A sensitivity analysis was performed to explore whether the distributions of Nutri-Score classifications in 2018 and 2020 were not limited to the diversification of foods (i.e. new foods to the market). For this analysis, identical foods available on the market in both 2018 and 2020 (thus foods of which the unique European Article Number was available in both years) were compared by calculating the distribution of Nutri-Score classifications.

The top 5 food groups with the largest change in percentage points over all Nutri-Score classifications were selected for further in-depth analysis in relation to food composition. For these selected food groups, frequency distributions were plotted to visually assess changes in frequencies in Nutri-Score classification over the final sum of points for 2018 and 2020. In addition, median contents were calculated for the nutrient contents that relatively changed the most between 2018 and 2020, for each Nutri-Score classification and by food group. Statistical analyses were carried out using SAS (version 9·4) and R (version 4·3·0).

## Results

A total of 38 295 foods in 2018 and 48 091 in 2020, categorised into 21 food groups, were included in the analyses. Food groups with the largest group size were bread (substitutes), such as rusks, knäckebröd, breadsticks (2018: *n* 4125; 2020: *n* 6984), cheeses (2018: *n* 4682, 2020: *n* 4183), baked goods and pastries (2018: *n* 4943; 2020: *n* 6248), and sweets and sweet goods such as candy, chocolate and ice cream (2018: *n* 6616; 2020: *n* 8767). For each food group, the median and interquartile range of energy and nutrient contents in 2018 and 2020 are shown in Table 1. For all food groups except for processed legumes, the median energy content changed in 2020 compared to 2018. Cheeses had a slightly higher median energy content, whereas other median nutrient contents of cheeses did not change. For meat preserves, besides the median energy content, the median SFA content was also lower. For other food groups, at least three median nutrient contents changed in 2020 compared to 2018. Breakfast cereals, dairy and plant-based solid foods, and meat substitutes were observed to have changes in all median nutrient contents in 2020 compared to 2018. Changes in median nutrient contents were both higher and lower and mostly include relatively small changes. However, a few observed changes were relatively large, such as the lower median sugar content of dairy and plant-based beverages (6·9 g per 100 g in 2018 *v*. 4·7 g per 100 g in 2020) and the lower median SFA content of sweets and sweet goods (4·8 g per 100 g in 2018 *v*. 4·2 g per 100 g in 2020).

**Table 1:** Median and IQR for energy and nutrient contents in 2018 and 2020 by food group

Food group	N		Energy content (kJ/100 g)				Salt content (g/100 g)				SFA content (g/100 g)				Sugar content (g/100 g)				Fibre content (g/100 g)				Protein content (g/100 g)			
	2018	2020	2018		2020		2018		2020		2018		2020		2018		2020		2018		2020		2018		2020	
			Median	IQR	Median	IQR	Median	IQR	Median	IQR	Median	IQR	Median	IQR	Median	IQR	Median	IQR	Median	IQR	Median	IQR	Median	IQR	Median	IQR
Vegetable preserves	1161	1058	*188*	*196*	*181*	*200*	0·40	0·43	0·40	0·53	0·10	0·30	0·10	0·30	*2·8*	*4·3*	*3·0*	*4·8*	*2·6*	*1·3*	*2·8*	*1·04*	1·6	2·0	1·6	1·9
Fruit preserves	596	605	*293*	*88*	*280*	*102*	0·01	0·03	0·01	0·03	0	0·05	0	0·08	*15*	*4·0*	*14*	*5·0*	*1·2*	*0·35*	*1·3*	*0·30*	0·40	0·20	0·40	0·20
Processed legumes	247	302	439	79	439	74	*0·40*	*0·3*	*0·30*	*0·3*	*0·20*	*0·20*	*0·10*	*0·10*	*1·0*	*1·3*	*0·70*	*1·1*	*6·5*	*2·0*	*6·0*	*2·0*	*6·6*	*1·8*	*6·8*	*2·2*
Bread (substitutes)	4125	6894	*1121*	*428*	*1084*	*286*	1·0	0·3	1·0	0·3	0·70	1·2	0·70	0·90	2·2	4·0	2·2	3·8	*3·5*	*3·2*	*3·7*	*3·2*	*9·0*	*3·1*	*9·2*	*3·0*
Breakfast cereals	498	634	*1626*	*260*	*1663*	*254*	*0·10*	*0·32*	*0·09*	*0·25*	*1·5*	*2·9*	*1·8*	*3·0*	*16*	*13*	*13*	*12*	*8·3*	*4·0*	*8·7*	*3·8*	*9·8*	*2·5*	*10*	*3·4*
Dairy and plant-based solid foods	1432	1523	*397*	*213*	*399*	*241*	*0·13*	*0·08*	*0·12*	*0·05*	1·9	2·5	1·9	2·8	*12*	*5·8*	*11*	*6·5*	*0·45*	*0*	*0·67*	*0*	3·4	1·8	3·4	1·9
Dairy and plant-based beverages	328	366	*213*	*107*	*191*	*104*	*0·10*	*0·02*	*0·11*	*0·02*	0·10	0·45	0·10	0·50	*6·9*	*7·3*	*4·7*	*4·7*	*0·26*	*0·40*	*0·37*	*0·20*	*2·6*	*1·1*	*2·9*	*1·6*
Cheeses	4682	4183	*1561*	*309*	*1573*	*327*	1·8	0·60	1·8	0·50	21	5·0	21	5·0	0	0·10	0	0·1	0	0	0	0	25	5·0	25	5·0
Meat preparations	1482	2497	*900*	*381*	*942*	*370*	1·3	1·0	1·3	0·78	*4·6*	*5·2*	*5·0*	*5·1*	*0·50*	*0·80*	*0·44*	*0·87*	*0·50*	*0·50*	*0·59*	*0·50*	*17*	*5·0*	*16*	*4·0*
Cold-cut meats	2607	2726	*1180*	*695*	*1169*	*685*	2·4	1·2	2·4	1·3	*8·8*	*7·0*	*8·5*	*7·4*	0·70	0·70	0·70	0·6	*0·10*	*0·30*	*0·20*	*0·40*	*17*	*8·0*	*18*	*8·0*
Meat preserves	502	593	*900*	*490*	*859*	*596*	1·9	0·60	1·9	0·70	*6·1*	*5·6*	*5·7*	*6·9*	0·50	1·1	0·50	2·2	0·37	0·95	0·37	0·77	13	3·0	13	3·0
Meat substitutes	255	640	*778*	*289*	*844*	*320*	*1·5*	*0·60*	*1·4*	*0·60*	*1·3*	*1·7*	*1·4*	*2·35*	*1·8*	*2·5*	*1·6*	*1·8*	*4·0*	*2·9*	*4·1*	*2·4*	*13*	*11*	*14*	*8·7*
Soups	1068	1218	*172*	*125*	*181*	*120*	*0·80*	*0·14*	*0·79*	*0·22*	0·40	0·70	0·40	0·8	1·2	2·1	1·2	2·0	*0·70*	*0·50*	*0·80*	*0·36*	*1·1*	*1·3*	*1·2*	*1·2*
Sauces	2077	2502	*770*	*1142*	*806*	*1075*	*1·4*	*0·93*	*1·5*	*0·90*	1·5	4·3	1·5	3·9	*6·5*	*11*	*6·9*	*13*	0·80	1·2	0·80	1·3	1·2	1·1	1·2	0·90
Hot savoury snacks	699	755	*983*	*460*	*988*	*457*	1·3	0·50	1·3	0·40	*3·6*	*5·3*	*4·0*	*5·9*	2·4	3·3	2·4	3·0	1·5	0·70	1·5	0·54	*7·8*	*3·3*	*8·0*	*3·2*
Cold savoury snacks	1133	1302	*2121*	*289*	*2092*	*266*	1·7	0·90	1·7	0·90	*3·0*	*3·7*	*2·7*	*1·7*	*2·8*	*3·4*	*2·7*	*4·0*	*2·7*	*2·0*	*3·0*	*2·0*	*6·5*	*3·0*	*6·6*	*3·2*
Processed nuts and seeds	1342	1614	*2573*	*444*	*2535*	*435*	0·05	0·80	0·05	0·79	*6·3*	*3·4*	*6·2*	*3·4*	*5·3*	*5·0*	*5·0*	*4·1*	*6·4*	*2·5*	*6·8*	*2·2*	18	7·0	18	7·0
Baked goods and pastries	4943	6248	*1799*	*485*	*1756*	*566*	0·52	0·45	0·52	0·45	*9·7*	*7·5*	*9·6*	*7·6*	*31*	*13*	*30*	*14*	*1·7*	*2·0*	*1·6*	*1·6*	5·0	2·6	5·0	2·6
Sweets and sweet goods	6616	8767	*1573*	*921*	*1642*	*825*	*0·10*	*0·17*	*0·11*	*0·18*	*4·8*	*15·8*	*4·2*	*17*	52	33	52	29	*2·2*	*3·4*	*2·3*	*1·7*	*3·5*	*5·0*	*3·9*	*5·4*
Drinks	2106	3126	*96*	*129*	*81*	*124*	*0*	*0·02*	*0·01*	*0·03*	0	0	0	0	*5·0*	*8·0*	*4·5*	*7·5*	*0·01*	*0·01*	*0·02*	*0*	0	0	0	0
Pizzas	314	410	*962*	*138*	*935*	*144*	1·1	0·30	1·1	0·36	*3·5*	*1·6*	*3·4*	*1·6*	*3·0*	*1·6*	*2·6*	*1·5*	*2·0*	*0·30*	*1·7*	*0·70*	9·7	1·0	9·6	1·3

N = number of foods. IQR = interquartile range. Values shown in *italics* indicate differences between median contents of 2018 and 2020.

Table 2 shows the distribution of Nutri-Score classifications in 2018 and 2020 by food group and the changes between these years in percentage points per Nutri-Score classification. In 2018 as well as in 2020, foods within the food groups, except for processed legumes, would have covered a range of Nutri-Score classifications (at least three and up to five). Vegetable preserves, fruit preserves and processed legumes would have largely received a Nutri-Score classification A (73–99 %), whereas cold-cut meats, baked goods and pastries, and sweets and sweet goods would be largely classified with Nutri-Score classification E (64–75 %). The mean and distribution of the final sum of points at food group level in 2018 and 2020 are shown in see online supplementary material, Supplementary Table 2. The results of the sensitivity analysis for foods with identical European Article Numbers also show changes in the distribution of Nutri-Score classifications of foods that were sold in both 2018 and 2020, for example for cold savoury snacks and pizzas, 3 % of the foods in each food group changed in Nutri-Score classification, whereas this was 6 % for each of the two food groups in the calculations for all foods available on the market (Table 2, see online supplementary material, Supplementary Tables 3–4). Therefore, food reformulation in several food groups had occurred due to changes in food compositions of existing foods on the market, as well as due to the diversification of foods within food groups of 2018 and 2020, such as for breakfast cereals, meat preserves and sweets and sweet goods.

**Table 2:** Distribution of Nutri-Score classifications (A–E, in %) in 2018 and 2020 and changes between 2018 and 2020 per Nutri-Score classification (in percentage point) by food group

		Distribution of Nutri-Score classifications (%)															
		2018					2020						Change (percentage point) between 2018 and 2020				
Food group	N	A	B	C	D	E	N	A	B	C	D	E	A	B	C	D	E
Vegetable preserves	1161	79	9	7	3	2	1058	78	9	7	4	2	–1	0	0	1	0
Fruit preserves	596	73	14	5	5	3	605	75	12	5	5	3	2	–2	0	0	0
Processed legumes	247	99	0	1	0	0	302	99	0	0	0	0	0	0	–1	0	0
Bread (substitutes)	4125	15	14	42	21	8	6894	17	16	43	18	6	2	2	1	–3	–2
**Breakfast cereals**	**498**	**31**	**9**	**33**	**25**	**2**	**634**	**35**	**11**	**32**	**20**	**3**	**4**	**2**	**–1**	**–5**	**1**
**Dairy and plant-based solid foods**	**1432**	**10**	**18**	**55**	**14**	**3**	**1523**	**15**	**18**	**51**	**10**	**5**	**5**	**0**	**–4**	**–4**	**2**
**Dairy and plant-based beverages** *	**328**	**0**	**20**	**45**	**6**	**29**	**366**	**0**	**33**	**39**	**12**	**16**	**0**	**13**	**–6**	**6**	**–13**
Cheeses	4682	0	0	2	88	9	4183	0	0	3	87	10	0	0	1	–1	1
Meat preparations	1482	10	11	17	47	16	2497	10	10	18	50	12	0	–1	1	3	–4
Cold-cut meats	2607	1	1	2	28	68	2726	1	1	4	30	64	0	0	2	2	–4
**Meat preserves**	**502**	**1**	**4**	**10**	**45**	**39**	**593**	**2**	**5**	**18**	**38**	**37**	**1**	**1**	**8**	**–7**	**–2**
Meat substitutes	255	25	17	30	24	4	640	28	14	33	23	2	3	–3	3	–1	–2
Soups	1068	4	36	59	0	1	1218	4	38	55	2	1	0	2	–4	2	0
Sauces	2077	0	2	29	42	27	2502	0	1	26	45	27	0	–1	–3	3	0
Hot savoury snacks	699	0	1	34	48	17	755	0	3	28	50	18	0	2	–6	2	1
Cold savoury snacks	1133	0	0	8	51	41	1302	0	0	11	54	35	0	0	3	3	–6
Processed nuts and seeds	1342	33	15	39	12	1	1614	36	15	39	10	0	3	0	0	–2	–1
Baked goods and pastries	4943	0	0	5	21	74	6248	0	1	5	23	71	0	1	0	2	–3
**Sweets and sweet goods**	**6616**	**1**	**4**	**8**	**17**	**70**	**8767**	**1**	**6**	**6**	**13**	**75**	**0**	**2**	**–2**	**–4**	**5**
Drinks*	2106	0	8	33	26	34	3126	0	8	37	27	28	0	0	4	1	–6
Pizzas	314	0	0	50	50	1	410	0	1	55	44	0	0	1	5	–6	–1

N = number of foods. Rows shown in **bold** indicate the top 5 food groups with the largest overall change in Nutri-Score classifications within food groups in 2020 compared to 2018. These food groups are selected for further in-depth analysis.

* For beverages, only mineral waters receive a Nutri-Score classification A.

Overall, changes between 2018 and 2020 in percentage point for Nutri-Score classifications per food group were small compared to the top 5 food groups with the largest overall change (≥7 percentage points change). The top 5 food groups were breakfast cereals, dairy and plant-based solid foods, dairy and plant-based beverages, meat preserves, and sweets and sweet goods. These food groups were selected for further in-depth analysis with respect to food composition.

**Fig. 1:**
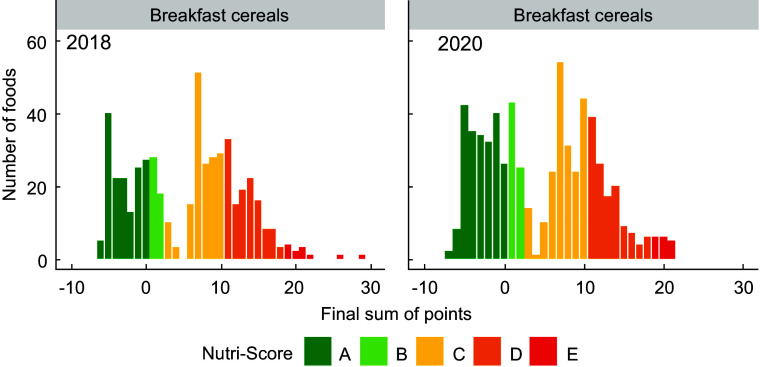
Frequency distribution of the final sum of points for breakfast cereals in 2018 (*n* 498) and 2020 (*n* 634)

**Fig. 2:**
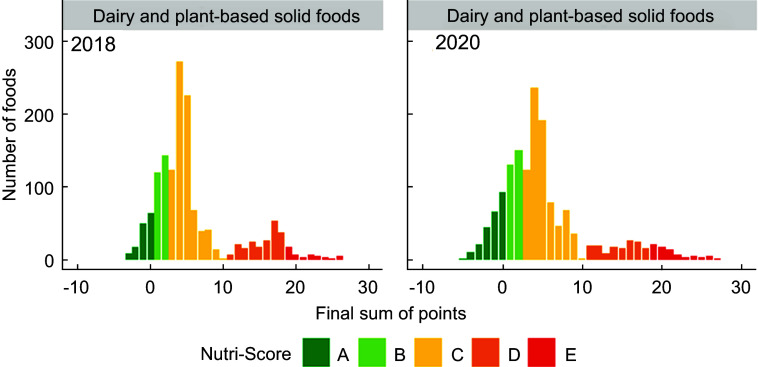
Frequency distribution of the final sum of points for dairy and plant-based solid foods (e.g. yoghurt, quark and desserts) in 2018 (*n* 1432) and 2020 (*n* 1523)

**Fig. 3:**
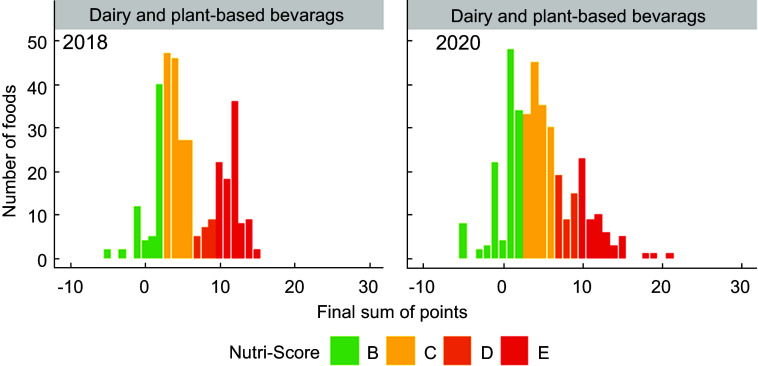
Frequency distribution of the final sum of points for dairy and plant-based beverages in 2018 (*n* 328) and 2020 (*n* 366). For beverages, only mineral waters receive a Nutri-Score classification A

**Fig. 4:**
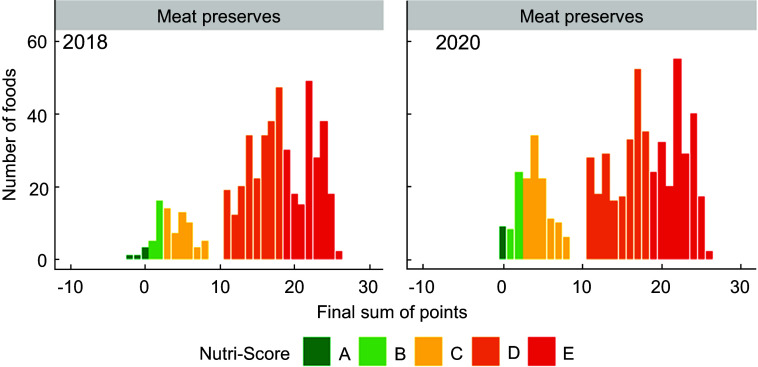
Frequency distribution of the final sum of points for meat preserves (e.g. Frankfurter and smoked sausages, hot dogs) in 2018 (*n* 502) and 2020 (*n* 593)

**Fig. 5:**
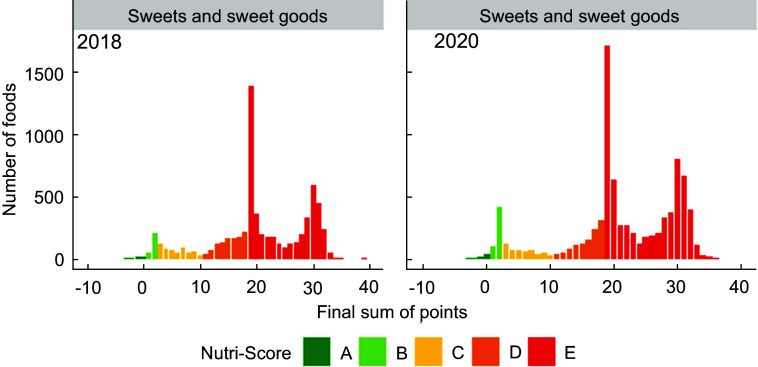
Frequency distribution of the final sum of points for sweets and sweet goods in 2018 (*n* 6616) and 2020 (*n* 8767)

Figures 1–5 show the visualisation of the changes in Nutri-Score classifications between 2018 and 2020 for the selected food groups. Changes in the frequency distribution of foods over the sum of points (bar height) and the corresponding Nutri-Score classifications (bar colour) were observed for all five of the selected food groups in 2020 compared to 2018. These changes occurred for Nutri-Score classifications A to E within all five food groups, except for dairy and plant-based beverages (Figs. 1–5).

For breakfast cereals, the proportions of foods that would receive Nutri-Score classifications A and B were higher (four and two percentage points respectively, see Table 2) in 2020 at the expense of the proportion of foods with Nutri-Score classifications C and D as these differed by six percentage points. The percentages of foods with Nutri-Score classifications A and E for dairy and plant-based solid foods were higher (five and two percentage points respectively) at the expense of Nutri-Score classifications C and D (difference of four percentage points each). Dairy and plant-based beverages had a higher proportion of Nutri-Score classifications B and D (13 and six percentage points respectively) at the expense of Nutri-Score classifications C and E (six and 13 percentage points respectively). Meat preserves included more Nutri-Score classifications A, B and C (ten percentage points in total) at the expense of Nutri-Score classifications D and E (nine percentage points in total). The percentages of foods with Nutri-Score classifications B and E for sweets and sweet goods went up by two and five percentage points respectively at the expense of Nutri-Score classifications C and D by two and four percentage points respectively (Table 2, Figs. 1–5).

For the selected food groups, favourable changes were observed for the median SFA contents of meat preserves and sweets and sweet goods, and the median sugar content of breakfast cereals, dairy and plant-based solid foods and dairy and plant-based beverages. For these food groups and nutrients, the median content within Nutri-Score classifications was calculated by food group (Table 3). Median SFA and sugar contents within Nutri-Score classifications were similar for 2018 and 2020: ascending from low median content in Nutri-Score classification A towards higher median content in Nutri-Score classification E. Median SFA content for meat preserves and sweets and sweet goods was relatively low in Nutri-Score classifications A, B and C compared to D and E, in both 2018 and 2020.

**Table 3:** Median sugar and SFA content per 100 g within Nutri-Score classifications in 2018 and 2020 of selected food groups

	Distribution within Nutri-Score classifications																			
	2018										2020									
	A		B		C		D		E		A		B		C		D		E	
Food group	N	P50	N	P50	N	P50	N	P50	N	P50	N	P50	N	P50	N	P50	N	P50	N	P50
*Sugar content (g per 100 g)*																				
Breakfast cereals	154	4·55	46	13·5	162	17·0	124	23·0	12	31·5	219	6·00	68	14·0	202	16·0	128	22·0	17	29·0
Dairy and plant-based solid foods	140	4·55	261	9·20	781	12·0	201	20·0	49	25·0	235	4·00	281	8·10	783	12·0	147	19·0	77	23·0
Dairy and plant-based beverages*	.	.	65	3·30	147	5·30	21	8·00	95	12·0	.	.	121	3·30	143	5·90	43	7·90	59	11·0
*SFA (g per 100 g)*																				
Meat preserves	5	0·90	21	1·20	52	1·70	226	5·40	198	10·0	9	1·00	32	1·20	105	1·40	228	5·15	219	10·0
Sweets and sweet goods	52	0·20	261	0·10	559	0·60	1100	5·40	4644	9·60	85	0·20	518	0·10	514	0·50	1117	3·30	6533	11·0

N = number of foods. P50 = 50^th^ percentile; median.

* For beverages, only mineral waters receive a Nutri-Score classification A.

## Discussion

The present study aimed to gain insight into food compositions and to calculate Nutri-Score classifications of foods sold in Dutch supermarkets in 2018 and 2020. Additionally, the present study aimed to investigate whether changes in the compositions of these foods could have led to different Nutri-Score classifications.

For the analyses in the present study, food compositions were used of branded foods that were available in Dutch supermarkets in 2018 and 2020. Foods with missing composition data were excluded from the analyses. Therefore, the results do not cover the whole Dutch retail market. However, the Dutch Branded Food database represents a large part (75 %) of the Dutch retail market^(20)^, and group sizes were considered sufficient, especially for bread (substitutes), cheeses, baked goods and pastries, and sweets and sweet goods. Limitations of the present study are that the mean fibre content of food groups was imputed to foods with missing fibre content and that estimations were applied for the fruits, vegetables and legumes component. Deviations from the actual median contents of food groups as well as the calculation of the Nutri-Score classifications of foods may therefore exist.

In the present study, changes were observed in the median energy and nutrient contents as well as in the calculated Nutri-Score classifications. These results indicate that food reformulation, whether it was the improvement of existing foods or the diversification of preexisting ones^(21)^, had taken place between 2018 and 2020. Although changes in nutrient contents of most food groups were small, a few of these changes were relatively large, which may have positively impacted the calculated Nutri-Score classification. However, improved Nutri-Score classifications were only observed for a small proportion of the food groups considered and for many nutrients and food group changes in nutrient contents were too small. For these food groups, larger reformulation efforts are needed in order to improve Nutri-Score classifications. In a recent study, larger reformulation efforts were assumed using theoretical scenario analyses with the previous Nutri-Score algorithm. A theoretical one-point reduction for the sodium (40 mg per 100 g or 0·1 g salt per 100 g), SFA (1·0 g per 100 g) or sugar (−1·5 g per 100 g in drinks and −4·5 g per 100 g in foods and dairy drinks per 100 g) content has shown to result in improved Nutri-Score classifications for several foods, such as for cereals, indicating that the improvement of food compositions to the extent of the reduction of points potentially results in improved Nutri-Score classifications^(17)^.

However, a theoretical one-point reduction for one of these components may not reflect the actual reformulation of foods by food manufacturers. The Nutri-Score algorithm takes into account a multitude of components, favourable and unfavourable, to which points are allocated. As seen in the present study, the median of several nutrient contents of many food groups remained unchanged or only slightly changed between 2018 and 2020. A reduction in just one unfavourable nutrient content as carried out in the theoretical analyses was observed for the sugar content of breakfast cereals and dairy and plant-based solid foods and beverages in the present study. Food reformulation may impact the amount of points that are allocated to nutrient contents and, therefore, may impact the final sum of points for foods, which is being translated to a Nutri-Score classification. However, in the present study, the allocation of points for the SFA contents for meat preserves and sweets and sweet goods is similar for foods with Nutri-Score classifications A, B and C. Thus, changes in the food composition do not necessarily result in a different Nutri-Score classification, especially since some of these changes were shown to be rather small or because favourable changes (for example decreased SFA content) may be compensated by unfavourable changes (such as increased sugar content).

Besides, a food’s Nutri-Score classification will not improve when the final sum of points stays within the threshold levels set by that Nutri-Score classification. The change in the final sum of points to warrant a change in Nutri-Score classification depends on the threshold levels set for the Nutri-Score classification. For example, a solid food, which is not an oil, fat, nut or seed, that is classified with a Nutri-Score classification C, has a final sum of points ranging from 3 to 10 points, whereas the thresholds for Nutri-Score classification B are 1–2 points. Because of the broader range of the final sum of points for Nutri-Score classification C, it may take larger reformulation efforts (a reduction of a maximum of 8 points) to improve the Nutri-Score classification compared to foods with Nutri-Score classification B. In case of breakfast cereals in the present study, a change from a median sugar content of 17 g per 100 g of Nutri-Score classification C to 13·5 g per 100 g of Nutri-Score classification B is translated to a reduction of two points. For beverages that are not mineral waters, however, a Nutri-Score classification B is the best Nutri-Score classification possible to receive because only mineral waters receive a Nutri-Score classification A. Reformulation of those beverages that are already classified as B are therefore not able to further improve in Nutri-Score classification^(13)^. Nevertheless, changes in Nutri-Score classifications are more likely to be observed for foods with a final sum of points closer to classification thresholds than foods with a final sum of points further away from those thresholds. Reformulation of foods with nutrient contents just below threshold levels was also found to be a strategy by food manufacturers in order to receive a more favourable classification on the label^(22,23)^.

Currently, only a limited number of studies have been carried out yet to examine the impact of a FOPNL on food reformulation. A recent review by Braesco & Drewnowski (2023) has found no studies on the impact of Nutri-Score on food reformulation. However, other FOPNL such as warning labels in Chile and the Health Star Rating in New-Zealand and Australia have resulted in the reduction in unfavourable nutrient contents (sugar, sodium, energy) and the increase in favourable nutrient contents (fibre) of foods^(24)^. Before the implementation of Nutri-Score in Belgium in 2018, significant differences were found between several nutrient contents of breakfast cereals in 2017 and 2018. Mean salt content was found to be significantly reduced by 20 % (0·1 g per 100 g) whereas other mean nutrients differed from –5·2 % to 2·2 %. Small changes in Nutri-Score classifications were observed for classifications B and D. It is uncertain, however, to what extent Nutri-Score had incentivised food manufacturers to reformulate foods as other commitments related to food reformulation were also in progress^(25)^. In several European countries, multiple reformulation strategies, for which targets are defined, are in place or planned^(26)^. For different FOPNL, it was found that sugar and salt were the most common nutrients that were targeted for reformulation^(23)^.

A limitation of the present study is that it was conducted in anticipation of the implementation of Nutri-Score in the Netherlands. Manufacturers of foods in the Dutch retail market were most likely not incentivised by Nutri-Score from 2018 to 2020 to reformulate foods since the implementation of Nutri-Score in the Netherlands was yet to be announced in 2023. The National Agreement to Improve Product Composition (2014–2020) was active during 2018 and 2020, although agreements were limited to specific types of foods^(3)^ and not all of these agreements led to improvements in food composition^(4)^. In the National Approach to Product Improvement, criteria are set up for broader food groups. These were published in 2022 and, therefore, may have had a limited impact on the reformulation efforts by food manufacturers from 2018 to 2020. Hence, food reformulation efforts during these years, and within the food group categorisation that was used, were limited to some food groups, such as breakfast cereals, meat preserves, sweets and sweet goods, and dairy and plant-based solid foods and beverages. For these groups, the present study observed changes in food composition between 2018 and 2020 resulting in different Nutri-Score classifications. In order to establish improvements in food composition and Nutri-Score classification, monitoring is recommended^(27)^. Results from monitoring studies may also detect elements for further improvement of the Nutri-Score algorithm, which may increase the incentivisation of food manufacturers to reformulate foods. Studies on this subject are recommended to also be conducted after the implementation of Nutri-Score in order to confirm its potential and to establish its extent to be an incentive for food reformulation to food manufacturers.

## Conclusions

From 2018 to 2020, Nutri-Score was not yet announced nor implemented in the Netherlands. During this period, reformulation efforts were not substantial. For food groups in the present study, similar median energy and nutrient contents and some differences in Nutri-Score classifications were observed. Using Nutri-Score as a FOPNL may encourage food manufacturers to increase their reformulation efforts in order to apply a more favourable Nutri-Score classification on the food packaging. Repeated monitoring and comparison of food compositions and Nutri-Score classifications is recommended in order to establish reformulation efforts by food manufacturers.

## Supporting information

Steenbergen and Temme supplementary material Steenbergen and Temme supplementary material

## Supplementary material

For supplementary material accompanying this paper visit https://doi.org/10.1017/S136898002400154X
