# Supplementary material for: Comparison of the nutritional composition and calculated Nutri-score classifications of the Dutch food retail supply in 2018 and 2020
Source: Public Health Nutr. 2024 Oct 10;27(1):e204. doi: 10.1017/S136898002400154X (PMC11604331; doi:10.1017/S136898002400154X)
Supplement: Steenbergen and Temme supplementary material [file S136898002400154Xsup001.docx]

**Supplementary material**

Supplementary Table 1. Food group categorization with mean fiber content in 2018 and 2020 and estimations for the percentage of fruits, vegetables and legumes.

|  |  | Fiber content (g/100 g) | | | | | | Estimation of fruit, vegetable and legumes content (%) |
| --- | --- | --- | --- | --- | --- | --- | --- | --- |
|  |  | 2018 | | | 2020 | | |  |
| Main food group | Sub food group | N_total_ | N | Mean | N_total_ | N | Mean |  |
| Baked goods and pastries | Biscuits | 642 | 547 | 3.2 | 634 | 521 | 4.0 | ≤40 |
|  | Cakes | 692 | 553 | 1.3 | 1037 | 877 | 1.3 | ≤40 |
|  | Cereal, muesli, fruit and energy bars | 145 | 141 | 8.9 | 226 | 221 | 8.6 | ≤40 |
|  | Shortbread cookies | 1239 | 1047 | 3.0 | 1333 | 1024 | 2.1 | ≤40 |
|  | Gingerbread | 284 | 263 | 4.5 | 358 | 333 | 5.7 | ≤40 |
|  | Pies and pastries | 1545 | 1390 | 1.3 | 2152 | 1919 | 1.3 | ≤40 |
|  | Waffles | 396 | 329 | 1.7 | 508 | 373 | 2.2 | ≤40 |
| Bread (substitutes) | Bases (pie, shell, crust) | 231 | 191 | 2.4 | 261 | 190 | 3.1 | ≤40 |
|  | Bread | 2365 | 2304 | 4.0 | 4476 | 4338 | 4.0 | ≤40 |
|  | Bread luxury, savoury | 175 | 161 | 2.3 | 287 | 265 | 2.4 | ≤40 |
|  | Bread luxury, plain and sweet | 508 | 486 | 3.2 | 834 | 804 | 3.3 | ≤40 |
|  | Bread substitutes | 846 | 706 | 5.9 | 1036 | 841 | 6.2 | ≤40 |
| Breakfast cereals | Breakfast cereals | 498 | 498 | 8.1 | 634 | 626 | 8.7 | ≤40 |
| Cheeses | Cheeses, semi-hard and hard | 3659 | 3109 | 0.0 | 3162 | 2451 | 0.0 | ≤40 |
|  | Cheeses, spreads and melting | 99 | 54 | 0.0 | 121 | 68 | 0.0 | ≤40 |
|  | Cheeses, soft | 924 | 515 | 0.3 | 900 | 513 | 0.3 | ≤40 |
| Cold savoury snacks | Crisps, sliced | 290 | 216 | 4.4 | 314 | 229 | 4.3 | ≤40 |
|  | Crisps, formed | 544 | 459 | 2.6 | 699 | 541 | 3.0 | ≤40 |
|  | Savoury cookies/biscuits/crackers | 299 | 250 | 2.5 | 289 | 179 | 2.7 | ≤40 |
| Cold-cut meats | Single processed meat, prepared | 491 | 395 | 0.1 | 601 | 351 | 0.1 | ≤40 |
|  | Single processed meat, raw/cured | 381 | 298 | 0.1 | 343 | 127 | 0.1 | ≤40 |
|  | Composed processed meat, prepared | 912 | 717 | 0.4 | 873 | 470 | 0.4 | ≤40 |
|  | Composed processed meat, raw/cured | 823 | 660 | 0.2 | 909 | 462 | 0.3 | ≤40 |
| Dairy and plant-based beverages | Dairy beverages | 250 | 151 | 0.3 | 248 | 102 | 0.4 | ≤40 |
|  | Plant-based beverages | 78 | 70 | 0.5 | 118 | 111 | 0.5 | ≤40 |
| Dairy and plant-based solid foods | Dairy solid foods | 1348 | 455 | 0.5 | 1343 | 372 | 0.7 | ≤40 |
|  | Plant-based solid foods | 84 | 61 | 0.8 | 180 | 129 | 1.0 | ≤40 |
| Drinks | Energy drinks | 98 | 39 | 0.0 | 159 | 43 | 0.0 | ≤40 |
|  | Soft drinks (ready to drink) | 1922 | 704 | 0.0 | 2816 | 905 | 0.0 | ≤40 |
|  | Sports drink (ready to drink) | 86 | 24 | 0.0 | 151 | 36 | 0.0 | ≤40 |
| Fruit preserves | Fruit preserves (processed) | 596 | 424 | 1.2 | 605 | 402 | 1.3 | >80 |
| Hot savoury snacks | Savoury snack breaded ragout | 217 | 89 | 1.3 | 221 | 59 | 1.3 | ≤40 |
|  | Savoury snack spring roll | 71 | 40 | 2.8 | 91 | 40 | 2.7 | ≤40 |
|  | Savoury snack meat | 288 | 214 | 1.6 | 282 | 183 | 1.5 | ≤40 |
|  | Savoury snack fried or puff pastry bun (no meat) | 123 | 75 | 1.7 | 161 | 73 | 1.8 | ≤40 |
| Meat preparations | Meat preparations, prepared | 107 | 74 | 1.0 | 61 | 38 | 1.0 | ≤40 |
|  | Meat preparations, unprepared | 1375 | 1134 | 0.6 | 2436 | 1702 | 0.6 | ≤40 |
| Meat preserves | Smoked sausage | 137 | 98 | 0.0 | 168 | 113 | 0.1 | ≤40 |
|  | Meat dish with sauce | 157 | 102 | 1.0 | 243 | 116 | 0.9 | ≤40 |
|  | Meat preserves | 208 | 137 | 0.4 | 182 | 84 | 0.4 | ≤40 |
| Meat substitutes | Meat substitutes | 255 | 237 | 4.0 | 640 | 560 | 4.1 | >40 |
| Pizzas | Pizzas | 314 | 223 | 2.0 | 410 | 294 | 1.7 | ≤40 |
| Processed legumes | Legumes processed | 247 | 241 | 6.5 | 302 | 287 | 6.0 | >80 |
| Processed nuts and seeds | Nuts and seeds | 1141 | 1049 | 6.7 | 1391 | 1171 | 7.1 | ≤40 |
|  | Coated nuts | 201 | 190 | 4.6 | 223 | 193 | 4.3 | ≤40 |
| Sauces | Cold sauces, tomato/vegetable base | 596 | 288 | 1.9 | 751 | 295 | 1.8 | ≤40 |
|  | Emulsified sauces | 850 | 363 | 0.5 | 1071 | 354 | 0.5 | ≤40 |
|  | Cooking sauces, tomato/vegetable base | 253 | 197 | 1.3 | 259 | 198 | 1.3 | >40 |
|  | Cooking sauces, other | 159 | 106 | 1.4 | 160 | 69 | 0.5 | ≤40 |
|  | Cooking sauces, oriental | 219 | 148 | 0.8 | 261 | 126 | 0.9 | ≤40 |
| Soups | Soups | 1068 | 848 | 0.8 | 1218 | 893 | 0.9 | >40 |
| Sweets and sweet goods | Chocolate | 2311 | 1039 | 4.1 | 3330 | 1485 | 3.7 | ≤40 |
|  | Ice cream | 1260 | 626 | 0.7 | 1144 | 509 | 1.0 | ≤40 |
|  | Candy | 3045 | 826 | 2.2 | 4293 | 701 | 2.3 | ≤40 |
| Vegetable preserves | Vegetable preserves (no pickles) | 1161 | 1057 | 3.0 | 1058 | 858 | 3.0 | >80 |

N_total_ = number of all foods available. N = number of foods of which fiber content is available.

Supplementary Table 2. Distribution of the final sum of points that correspond to Nutri-Score classifications by food group in 2018 and 2020.

| Food group |  | Final Nutri-Score points | | | | | | | | | | | | |
| --- | --- | --- | --- | --- | --- | --- | --- | --- | --- | --- | --- | --- | --- | --- |
|  |  | 2018 | | | | | |  | 2020 | | | | | |
|  | N | Mean | P5 | P25 | P50 | P75 | P95 | N | Mean | P5 | P25 | P50 | P75 | P95 |
| Vegetable preserves | 1161 | -2 | -8 | -4 | -3 | -1 | 11 | 1058 | -1 | -7 | -5 | -3 | 0 | 15 |
| Fruit preserves | 596 | 1 | -3 | -2 | -1 | 1 | 13 | 605 | 0 | -3 | -2 | -1 | 0 | 13 |
| Processed legumes | 247 | -8 | -11 | -10 | -9 | -7 | -3 | 302 | -8 | -11 | -10 | -8 | -7 | -4 |
| Bread (substitutes) | 4125 | 7 | -2 | 2 | 4 | 12 | 20 | 6894 | 6 | -2 | 1 | 4 | 10 | 19 |
| Breakfast cereals | 498 | 6 | -5 | -1 | 7 | 11 | 16 | 634 | 5 | -5 | -1 | 6 | 10 | 16 |
| Dairy and plant-based solid foods | 1432 | 6 | -1 | 2 | 4 | 6 | 18 | 1523 | 5 | -2 | 2 | 4 | 6 | 19 |
| Dairy and plant-based beverages | 328 | 6 | 0 | 3 | 5 | 10 | 13 | 366 | 5 | -1 | 2 | 4 | 7 | 12 |
| Cheeses | 4682 | 16 | 13 | 14 | 15 | 17 | 19 | 4183 | 16 | 12 | 14 | 15 | 17 | 19 |
| Meat preparations | 1482 | 11 | -2 | 4 | 13 | 17 | 24 | 2497 | 11 | -1 | 4 | 13 | 16 | 21 |
| Cold-cut meats | 2607 | 22 | 11 | 17 | 22 | 28 | 35 | 2726 | 22 | 8 | 16 | 22 | 28 | 35 |
| Meat preserves | 502 | 16 | 2 | 13 | 17 | 22 | 24 | 593 | 15 | 2 | 11 | 17 | 21 | 24 |
| Meat substitutes | 255 | 6 | -4 | 0 | 3 | 11 | 18 | 640 | 5 | -4 | 0 | 4 | 11 | 16 |
| Soups | 1068 | 3 | 1 | 2 | 3 | 3 | 5 | 1218 | 3 | 1 | 2 | 3 | 3 | 5 |
| Sauces | 2077 | 14 | 4 | 9 | 14 | 19 | 27 | 2502 | 15 | 4 | 10 | 14 | 19 | 27 |
| Hot savoury snacks | 699 | 12 | 4 | 7 | 12 | 17 | 22 | 755 | 13 | 4 | 8 | 13 | 17 | 21 |
| Cold savoury snacks | 1133 | 18 | 9 | 13 | 16 | 23 | 29 | 1302 | 17 | 9 | 12 | 16 | 21 | 28 |
| Processed nuts and seeds | 1342 | 1 | -9 | -7 | 3 | 8 | 13 | 1614 | 1 | -10 | -7 | 2 | 7 | 13 |
| Baked goods and pastries | 4943 | 22 | 10 | 18 | 23 | 27 | 29 | 6248 | 22 | 9 | 17 | 23 | 26 | 30 |
| Sweets and sweet goods | 6616 | 20 | 3 | 17 | 19 | 29 | 31 | 8767 | 21 | 2 | 18 | 20 | 29 | 32 |
| Drinks | 2106 | 7 | 0 | 4 | 8 | 11 | 13 | 3126 | 7 | 0 | 4 | 7 | 10 | 12 |
| Pizzas | 314 | 9 | 5 | 6 | 11 | 13 | 14 | 410 | 9 | 4 | 6 | 7 | 12 | 14 |

N = number of foods. P5 = 5^th^ percentile. P25 = 25^th^ percentile. P50 = 50^th^ percentile. P75 = 75^th^ percentile. P95 = 95^th^ percentile.

Supplementary Table 3. Distribution of Nutri-Score classifications (A-E, in %) in 2018 and 2020 and changes between 2018 and 2020 per Nutri-Score classification (in percentage point) by food group of foods that were available on the market in both 2018 and 2020 (identified by identical European Article Number).

|  |  | Distribution of Nutri-Score classifications (%) | | | | | | | | | | | | Change (percentage point) between 2018 and 2020 | | | | |
| --- | --- | --- | --- | --- | --- | --- | --- | --- | --- | --- | --- | --- | --- | --- | --- | --- | --- | --- |
|  |  | 2018 | | | | | 2020 | | | | | | |  |  |  |  |  |
| Food group | N | A | B | C | D | E | | A | B | C | D | E | A | | B | C | D | E |
| Vegetable preserves | 371 | 76 | 12 | 6 | 4 | 3 | | 76 | 12 | 6 | 4 | 3 | 0 | | 0 | 0 | 0 | 0 |
| Fruit preserves | 262 | 74 | 13 | 4 | 6 | 3 | | 74 | 13 | 4 | 6 | 3 | 0 | | 0 | 0 | 0 | 0 |
| Processed legumes | 93 | 98 | 1 | 1 | 0 | 0 | | 98 | 1 | 1 | 0 | 0 | 0 | | 0 | 0 | 0 | 0 |
| Bread (substitutes) | 2380 | 13 | 14 | 45 | 20 | 8 | | 14 | 15 | 43 | 20 | 8 | -1 | | -1 | 2 | 0 | 0 |
| Breakfast cereals | 199 | 37 | 10 | 28 | 21 | 4 | | 38 | 10 | 27 | 22 | 4 | -1 | | 0 | 1 | -1 | 0 |
| Dairy and plant-based solid foods | 666 | 12 | 17 | 59 | 9 | 3 | | 12 | 18 | 58 | 9 | 3 | 0 | | -1 | 1 | 0 | 0 |
| Dairy and plant-based beverages* | 163 | 0 | 45 | 39 | 2 | 14 | | 0 | 44 | 39 | 4 | 14 | 0 | | 1 | 0 | -2 | 0 |
| Cheeses | 2181 | 0 | 0 | 3 | 87 | 10 | | 0 | 0 | 3 | 88 | 9 | 0 | | 0 | 0 | -1 | 1 |
| Meat preparations | 456 | 10 | 12 | 13 | 48 | 18 | | 10 | 12 | 13 | 48 | 18 | 0 | | 0 | 0 | 0 | 0 |
| Cold-cut meats | 762 | 1 | 1 | 3 | 29 | 67 | | 1 | 1 | 3 | 29 | 67 | 0 | | 0 | 0 | 0 | 0 |
| Meat preserves | 292 | 1 | 4 | 16 | 40 | 39 | | 1 | 4 | 15 | 40 | 39 | 0 | | 0 | 1 | 0 | 0 |
| Meat substitutes | 49 | 20 | 12 | 39 | 27 | 2 | | 20 | 10 | 39 | 29 | 2 | 0 | | 2 | 0 | -2 | 0 |
| Soups | 471 | 4 | 32 | 63 | 1 | 1 | | 4 | 32 | 63 | 1 | 1 | 0 | | 0 | 0 | 0 | 0 |
| Sauces | 1016 | 0 | 1 | 26 | 47 | 25 | | 0 | 1 | 25 | 48 | 25 | 0 | | 0 | 1 | -1 | 0 |
| Hot savoury snacks | 330 | 0 | 1 | 24 | 54 | 21 | | 0 | 2 | 24 | 52 | 22 | 0 | | -1 | 0 | 2 | -1 |
| Cold savoury snacks | 468 | 0 | 0 | 7 | 51 | 42 | | 0 | 0 | 7 | 54 | 39 | 0 | | 0 | 0 | -3 | 3 |
| Processed nuts and seeds | 529 | 37 | 11 | 40 | 12 | 0 | | 37 | 11 | 40 | 12 | 0 | 0 | | 0 | 0 | 0 | 0 |
| Baked goods and pastries | 2696 | 0 | 1 | 4 | 24 | 72 | | 0 | 1 | 4 | 24 | 72 | 0 | | 0 | 0 | 0 | 0 |
| Sweets and sweet goods | 3611 | 2 | 7 | 6 | 31 | 55 | | 2 | 7 | 5 | 30 | 56 | 0 | | 0 | 0 | 1 | -1 |
| Drinks* | 1241 | 0 | 32 | 30 | 15 | 23 | | 0 | 32 | 30 | 15 | 22 | 0 | | 0 | 0 | 0 | 1 |
| Pizzas | 145 | 0 | 0 | 37 | 62 | 1 | | 0 | 1 | 39 | 59 | 1 | 0 | | -1 | -2 | 3 | 0 |

N = number of foods. * = for beverages, only mineral waters receive a Nutri-Score classification A.

Supplementary Table 4. Distribution of the final sum of points that correspond to Nutri-Score classifications by food group of foods that were available on the market in both 2018 and 2020 (identified by identical European Article Number)..

| Food group |  | Final Nutri-Score points | | | | | | | | | | | |
| --- | --- | --- | --- | --- | --- | --- | --- | --- | --- | --- | --- | --- | --- |
|  |  | 2018 | | | | | | 2020 | | | | | |
|  | N | Mean | P5 | P25 | P50 | P75 | P95 | Mean | P5 | P25 | P50 | P75 | P95 |
| Vegetable preserves | 371 | -1 | -7 | -5 | -3 | 0 | 15 | -1 | -7 | -5 | -3 | 0 | 15 |
| Fruit preserves | 262 | 1 | -2 | -2 | -1 | 1 | 13 | 1 | -3 | -2 | -1 | 1 | 13 |
| Processed legumes | 93 | -8 | -11 | -10 | -9 | -7 | -2 | -8 | -11 | -10 | -9 | -7 | -2 |
| Bread (substitutes) | 2380 | 7 | -2 | 2 | 5 | 12 | 20 | 7 | -2 | 2 | 5 | 12 | 20 |
| Breakfast cereals | 199 | 5 | -5 | -2 | 3 | 10 | 17 | 5 | -5 | -2 | 3 | 11 | 17 |
| Dairy and plant-based solid foods | 666 | 5 | -1 | 2 | 4 | 6 | 17 | 5 | -1 | 2 | 4 | 6 | 17 |
| Dairy and plant-based beverages | 163 | 5 | -1 | 2 | 4 | 6 | 12 | 4 | -1 | 2 | 4 | 6 | 12 |
| Cheeses | 2181 | 15 | 12 | 14 | 15 | 17 | 19 | 15 | 12 | 14 | 15 | 17 | 19 |
| Meat preparations | 456 | 12 | -2 | 5 | 14 | 17 | 24 | 12 | -2 | 4 | 14 | 17 | 23 |
| Cold-cut meats | 762 | 22 | 11 | 17 | 22 | 29 | 35 | 22 | 11 | 17 | 22 | 29 | 35 |
| Meat preserves | 292 | 16 | 3 | 12 | 17 | 21 | 24 | 15 | 2 | 12 | 17 | 21 | 24 |
| Meat substitutes | 49 | 7 | -4 | 2 | 4 | 11 | 18 | 7 | -4 | 2 | 4 | 11 | 18 |
| Soups | 471 | 3 | 1 | 2 | 3 | 3 | 5 | 3 | 1 | 2 | 3 | 3 | 5 |
| Sauces | 1016 | 15 | 4 | 10 | 14 | 19 | 26 | 14 | 4 | 10 | 14 | 18 | 26 |
| Hot savoury snacks | 330 | 13 | 5 | 11 | 13 | 18 | 22 | 13 | 5 | 10 | 13 | 18 | 22 |
| Cold savoury snacks | 468 | 18 | 10 | 12 | 17 | 23 | 29 | 18 | 10 | 13 | 17 | 21 | 29 |
| Processed nuts and seeds | 529 | 1 | -10 | -7 | 3 | 8 | 13 | 1 | -10 | -7 | 3 | 8 | 13 |
| Baked goods and pastries | 2696 | 22 | 11 | 18 | 23 | 27 | 30 | 22 | 11 | 18 | 23 | 27 | 30 |
| Sweets and sweet goods | 3611 | 20 | 1 | 17 | 19 | 29 | 31 | 20 | 1 | 18 | 19 | 29 | 31 |
| Drinks | 1241 | 7 | 0 | 4 | 7 | 11 | 13 | 7 | 0 | 4 | 7 | 11 | 12 |
| Pizzas | 145 | 10 | 5 | 7 | 11 | 13 | 15 | 10 | 5 | 6 | 11 | 13 | 15 |

N = number of foods. P5 = 5^th^ percentile. P25 = 25^th^ percentile. P50 = 50^th^ percentile. P75 = 75^th^ percentile. P95 = 95^th^ percentile.
